# Surgery for Glioblastoma: Impact of the Combined Use of 5-Aminolevulinic Acid and Intraoperative MRI on Extent of Resection and Survival

**DOI:** 10.1371/journal.pone.0131872

**Published:** 2015-06-26

**Authors:** Jan Coburger, Vincent Hagel, Christian Rainer Wirtz, Ralph König

**Affiliations:** Department of Neurosurgery, Campus Günzburg, University of Ulm, Ludwig Heilmeyerstr. 2, Günzburg, Germany; Johns Hopkins University, UNITED STATES

## Abstract

**Background:**

There is rising evidence that in glioblastoma(GBM) surgery an increase of extent of resection(EoR) leads to an increase of patient’s survival. Based on histopathological assessments tumor depiction of Gd-DTPA enhancement and 5-aminolevulinic-acid-fluorescence(5-ALA) might be synergistic for intraoperative resection control.

**Objective:**

To assess impact of additional use of 5-ALA in intraoperative MRI(iMRI) assisted surgery of GBMs on extent of resection(EoR), progression free survival(PFS) and overall survival(OS).

**Methods:**

We prospectively enrolled 33 patients with GBMs eligible for gross-total-resection(GTR) and performed a combined approach using 5-ALA and iMRI. As a control group, we performed a retrospective matched pair assessment, based on 144 patients with iMRI-assisted surgery. Matching criteria were, MGMT promotor methylation, recurrent surgery, eloquent location, tumor size and age. Only patients with an intended GTR and primary GBMs were included. We calculated Kaplan Mayer estimates to compare OS and PFS using the Log-Rank-Test. We used the T-test to compare volumetric results of EoR and the Chi-Square-Test to compare new permanent neurological deficits(nPND) and general complications between the two groups.

**Results:**

Median follow up was 31 months. No significant differences between both groups were found concerning the matching criteria. GTR was achieved significantly more often (p <0.010) using 5-ALA&iMRI (100%) compared to iMRI alone(82%). Mean EoR was significantly(p<0.004) higher in 5-ALA&iMRI-group(99.7%) than in iMRI-alone-group(97.4%) Rate of complications did not differ significantly between groups(21% iMRI-group,27%5-ALA&iMRI-group,p<0.518). nPND were found in 6% in both groups. Median PFS (6mo resp.;p<0.309) and median OS(iMRI:17mo;5-ALA&iMRI-group:18mo;p<0.708)) were not significantly different between both groups.

**Conclusion:**

We found a significant increase of EoR when combining 5-ALA&iMRI compared to use of iMRI alone. Maximizing EoR did not lead to an increase of complications or neurological deficits if used with neurophysiological monitoring in eloquent lesions. No final conclusion can be drawn whether a further increase of EoR benefits patient’s progression free survival and overall survival.

## Introduction

Most large studies show that extent of resection (EoR) is a key prognostic factor for patients harboring a glioblastoma (GBM). [1, 2] Best data concerning benefit for overall survival (OS) after increase of EoR are derived from the prospective randomized intraoperative imaging studies for 5-aminolevulinic acid (5-ALA). [3, 4]. Senft et al showed that use of low field iMRI leads to an increase in EoR and a benefit for PFS compared to white-light-resection without intraoperative imaging as a resection control. [5] Especially the introduction of high-field iMRI ideally enables the surgeon to resect the desired lesion completely while only being limited by eloquent areas. Thus, theoretically iMRI in combination with brain mapping is the ideal tool to establish the goal of a “maximum safe resection approach” as proclaimed by Marko et al.[6]. However, repetitive intraoperative Gd-DTPA administration leads to an unspecific oozing of contrast in the resection cavity.[7] Thus, it would be preferable to have another imaging technique like the above mentioned 5-ALA helping to achieve a near total resection before performing a “final” iMRI scan. Additionally, as several authors stated, the solid tumor boundaries in GBM exceed the Gd-DTPA enhancement in preoperative MRI.[8, 9] As shown in various PET-CT studies, tracer uptake in GBMs usually exceeds Gd-DTPA enhancement and seems to match with 5-ALA tissue fluorescence. [10] Our histological data confirms a difference in intraoperative solid tumor depiction using 5-ALA and Gd-DTPA enhanced iMRI. [11] Based only on this histological data 5-ALA should have a supplementary effect in a combined setup with iMRI. Thus, we performed a prospective study comparing patients with a combined imaging approach (5-ALA&iMRI) with a matched pair retrospective control group and evaluated EoR, clinical outcome, PFS and OS.

## Patients and Methods

### Patient’s selection criteria

We performed a retrospective assessment based on matched pairs of two cohorts. In the 5-ALA&iMRI cohort we prospectively included patients with a contrast enhancing lesion eligible for a gross total resection (GTR) and a final histopathological diagnosis of GBM WHO°IV from July 2012 to February 2014. Exclusion criteria were incomplete follow up or missing MGMT promotor methylation state. The control cohort (iMRI) was based on retrospective assessment of all patients who had iMRI assisted surgery from September 2008 to July 2012 before introduction of 5-ALA at our center to exclude a selection bias. Inclusion criteria for the control group were intended GTR, complete follow up and assessment of MGMT promotor methylation state. Matching criteria were tumor volume (< = 40cc or > 40cc), recurrent surgery, MGMT promotor methylation, eloquent location and age.

### OR Setup

All cases were performed in a dedicated 1.5 Tesla iMRI (Magnetom Espree, Siemens Healthcare, Erlangen, Germany) environment with integrated data management and neuronavigation (Brainsuite, Brainlab, Feldkirchen) running the Iplan 3.0 neuronavigation software (Brainlab, Feldkirchen, Germany). Application of 5-ALA was performed as described before.[3] We used a Zeiss Pentero 600 microscope with integrated head-up display for visualization of neuronavigation and a Blue 400 filter to perform 405nm fluorescence. Surgeries were performed by seven experienced neurosurgeons in an approximately equal share.

### Eloquent areas: Definition and surgical technique

Eloquent tumors were defined as tumors involving the motor or language system. Lesions infiltrating the visual cortex or optic radiation were not considered as eloquent and thus resected according to patients consent. Preoperative diagnostic work-up for eloquently located tumors included BOLD imaging and diffusion tensor imaging followed by deterministic fiber tracking (corticospinal tract, arcuate fascicle). Surgery for tumors adjacent to the motor cortex or corticospinal tract were performed applying intraoperative monitoring (MEP, direct cortical stimulation, subcortical stimulation). We did not perform awake surgery for language mapping in patients with suspected high grade gliomas.

### Intraoperative MRI: imaging protocol

Intraoperatively we routinely performed a T1 MPRAGE with and without Gd-DTPA and an axial T2 and Flair sequence. Timing of iMRI was at surgeon’s discretion, as well as the application of additional sequences. Usually in lesions close to important fiber tracts intraoperative DTI and fiber tracking was performed.

### Volumetric assessment

Volumetric assessment was performed using Iplan 3.0 Software (Brainlab, Feldkirchen, Germany). Volume measurement was based on preoperative and postoperative (<72h) Gd-DTPA enhancement.

### Definition of evaluated items

A lesion was defined as being ‘eloquent’ if it was located within or adjacent to an eloquent area thus potentially interfering with the resection of more than the contrast enhancing tumor. The location “other” was used for subcortical eloquent areas; like corpus callosum, basal ganglia and brainstem. An EoR >95% was defined as a GTR. Progression free survival (PFS) was defined as survival from last surgery until first radiological description of a progression. Follow up MRI was routinely performed every 3 months. Results in this study are based on the evaluation of the local tumor board review (Comprehensive Cancer Center Ulm (CCCU)). Decision making was based on the Response Assessment in Neuro-Oncology criteria (RANO) as actualized in 2010. [12] Overall survival (OS) was defined as survival after date of primary diagnosis in months. New permanent neurological deficits were defined as neurological deficits not described prior to surgery, which were still eminent at first follow up assessment (3months). MGMT promotor methylation was assessed and reported according to Hegi et al.[13]

Aim of surgery is routinely reported in the OR notes at our center. Whether a lesion was eligible for a GTR or not was decided and reported by the individual surgeon. Patients of the retrospective control cohort which were eligible for a GTR were selected based on an evaluation of these OR notes.

### Ethical approval

Ethical approval was received by the local ethical committee (Ethik Kommission Universität Ulm) with approval number 172/12. Patient data was pseudonymized before assessment and publication.

### Statistics

We used the Chi-Square test for nominal variables and Student-T-test for metric variables to compare iMRI alone vs. 5-ALA&iMRI. Only 2-sided p values were reported. Kaplan Meyer Charts of PFS and OS were calculated for all influencing variables. Differences were assessed using Log-Rank test. We used a reversed Kaplan-Meyer approach to calculate follow up.[14] A p value < 0.05 was defined as statistically significant. All calculations were done using SPSS 21.0 (Lead Technologies Inc., Charlotte, NC, USA).

## Results

49 patients met the inclusion criteria for a combined treatment (5-ALA&iMRI) according to the prospective study protocol. 11 patients were excluded based on final histopathology other than GBM. 2 patients were excluded since a secondary GBM was found. 2 patients had no MGMT promotor state and 1 patient was lost to follow up. 33 patients finally entered the matched pair assessment as part of the 5-ALA&iMRI cohort. Matched pairs of the control group were recruited from 144 patients who had surgery using iMRI alone.

In all patients we found a typical fluorescence of the tumor tissue. In two of 33 patients a residual fluorescence was seen and intentionally not resected. In one case it was located in ventricular ependyma, and in the other case an infiltration of the corpus callosum was found. In both cases an incomplete resection was confirmed in early postoperative MRI. In 5-ALA&iMRI group in 8 patients an incomplete resection was found in postoperative MRI. Intraoperative rate of residual fluorescence (6%) was lower than the postoperative rate of residual Gd-DTPA enhancement (24%).

Table 1 shows the distribution of the matching criteria eloquent location, MGMT promotor methylation, recurrent surgery and age. A similar distribution was achieved for MGMT promotor (methylated in 53%), recurrent surgery (19%) and categorical distribution of eloquent location (eloquent in 45%) and tumor size (>40cc in 39% of cases). Age was slightly lower in 5-ALA&iMRI group (57years vs 60years). The difference was not significant in T-test, however (p<0.251).

**Table 1:** distribution of matching criteria.

variables		iMRI	5-ALA & iMRI	Chi-Square/ T-test
mean age (range)		59 (29–77)	57 (26–76)	p < 0.251
std. error of mean		2.26	2.25	
tumor size	< = 40cc	20(61%)	20(61%)	p < 1.000
	> 40 cc	13(39%)	13(39%)	
pre-op tumor volume	cc	42.6	38.0	p < 0.605
std. error of mean		7.18	5.10	
MGMT promotor	negative	14(42%)	14(42%)	p < 1.000
	slightly positive	2(6%)	2(6%)	
	positive	17(52%)	17(52%)	
eloquent location	not eloquent	15 (46%)	15 (46%)	p < 1.000
	motor	6 (18%)	4 (12%)	
	language	8 (24%)	7 (21%)	
	other	4 (12%)	7 (21%)	
recurrent surgery	no	27 (82%)	27 (82%)	p < 1.000
	yes	6 (18%)	6 (18%)	

iMRI: intraoperative high field MRI; 5-ALA: 5-aminolevulinic acid; cc: cubic centimeter

**Fig 1:**
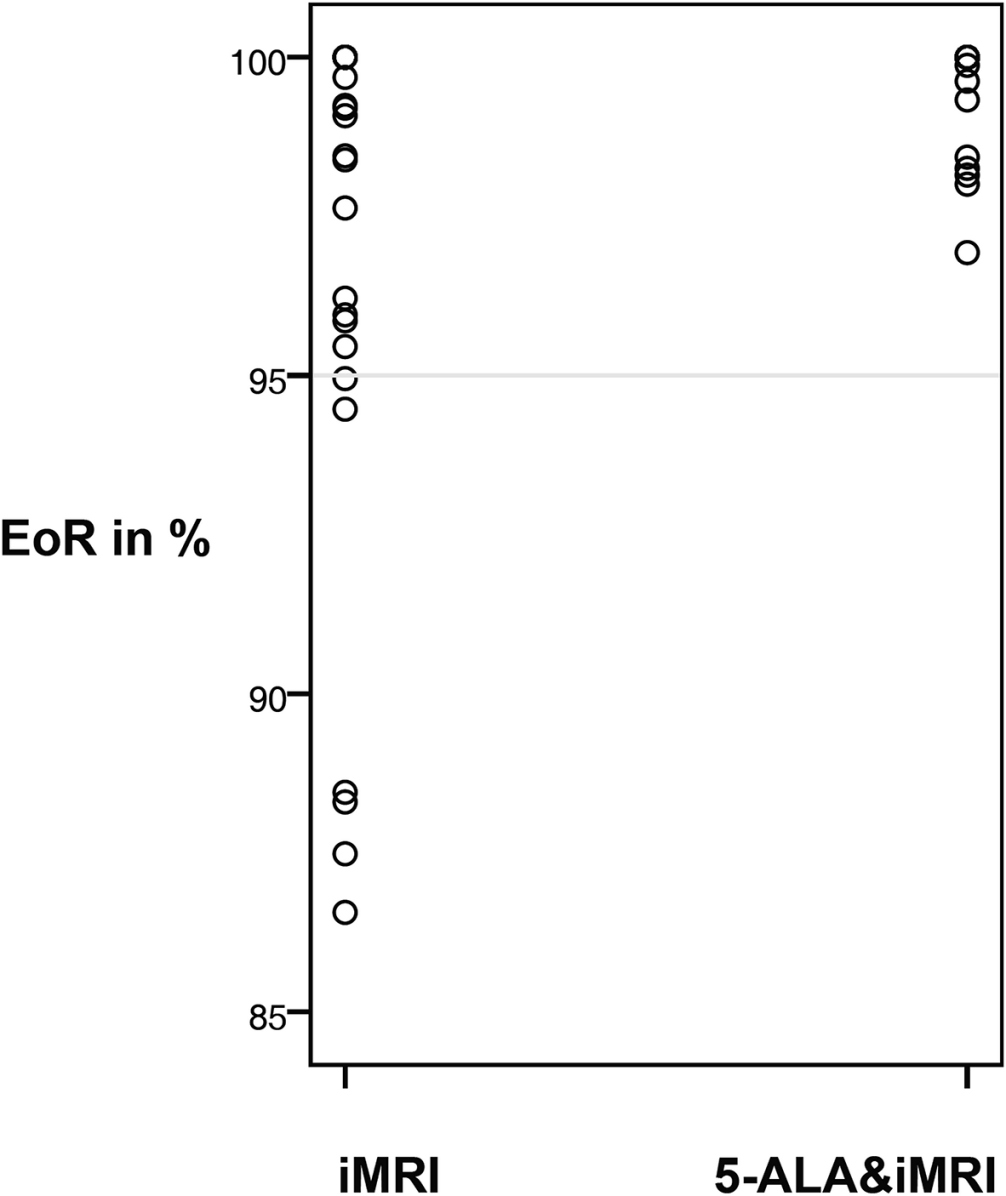
Scattered plot on extent of resection divided by iMRI group and 5-ALA&iMRI group. EoR: extent of resection, iMRI intraoperative MRI; 5-ALA: 5 Aminolevulinic acid.

Volumetric assessment is shown in Table 2. We found no significant differences in preoperative tumor volume between both groups (p < 0.605). Postoperative tumor volume was significantly (p < 0.017) smaller in 5-ALA&iMRI group (0.08cc) than in iMRI group (0.7cc). Similar results were found in the calculated EoR: Mean EoR was 99.7% in 5-ALA&iMRI group and 97.4% in iMRI group. The two groups significantly differed in this regard as well (p < 0.004). Fig 1 shows a scattered plot of the results of EoR in both groups. Using the combined imaging approach in all cases an EoR above 95% was reached. In iMRI group in 18% only an EoR below 95% was achieved. Minimum EoR was 87% in this group and 97% in 5-ALA&iMRI group. The difference in residual volume (iMRI 1.1cc vs. 5-ALA&iMRI 0.07cc, p < 0.026) and in mean EoR was slightly more pronounced in eloquent lesions (iMRI 96% vs. 5-ALA&iMRI 99.6%, p < 0.006). In recurrent disease residual volume is lower in 5-ALA&iMRI group (0.04cc vs. 0.34cc, p<0.227) and EoR slightly higher (99.2 vs. 99.0, p<0.877). However, no significant difference were found.

**Table 2:** Volumetric assessment.

variables		iMRI	5-ALA & iMRI	T-test
pre-op tumor volume	cc	42.6	38.0	p < 0.605
range		0.5–200.0	1.0–111.9	
std. error of mean		7.18	5.10	
post-op tumor volume	cc	0.70	0.08	p < 0.017
range		0–6.3	0–0.8	
std. error of mean		0.24	0.03	
EoR	%	97.4	99.7	p < 0.004
range		87–100	97–100	
std. error of mean		0.71	0.13	

iMRI: intraoperative high-field MRI; 5-ALA: 5-aminolevulinic acid; EoR: extent of resection

Surgical outcome is shown in Table 3. Median follow up was 26 months (CI95% 21–31) in 5-ALA&iMRI group and 42 months in iMRI group (CI95% 39–45). We found a significantly higher rate of GTR: 76% in the 5-ALA&iMRI-group compared to the iMRI-alone-group (100% vs 82%, p<0.022). No significant differences were found concerning complication rates in general (p<0.518). Rate of hemorrhage was slightly higher in 5-ALA&iMRI group (15% vs. 6%), however significant differences were not found (p<0.079). No difference was seen between rates of nPND (6%).

**Table 3:** Outcome of surgery.

variables		iMRI	5-ALA & iMRI	Chi-Square-/Log Rank-Test
EoR	GTR	27 (82%)	33 (100%)	p < 0.010
complications	none	26 (79%)	24 (73%)	p < 0.518
	csf leak	2 (6%)	1 (3%)	p < 0.157
	hemorrhage	1 (3%)	5 (15%)	p < 0.079
	infection	1 (3%)	1 (3%)	p < 0.982
	‚sun-burn‘	0 (0%)	1 (3%)	p < 0.306
	thrombosis	1 (3%)	1 (3%)	p < 0.321
	nPND	2 (6%)	2 (6%)	p < 0.975
median survival	PFS (CI95%)	6 (2.4–9.6)	6 (4.6–7.4)	p < 0.309
	OS (CI95%)	17 (7.6–26.4)	18 (15.2–20.8)	p < 0.708

iMRI: intraoperative high field MRI; 5-ALA: 5-aminolevulinic acid; GTR: gross total resection; nPND: new permanent neurological deficits; PFS progression free survival; OS overall survival; CI95%: 95% confidence interval

We performed a subgroup assessment of eloquent lesions only. 18 patients in both groups had surgery of an eloquent lesion. We found no significant difference between mean preoperative tumor volumes of iMRI and 5-ALA&iMRI group (45.5cc vs. 35.4cc, p<0.462). Postoperative residual tumor volume was significantly lower in 5-ALA&iMRI group compared to iMRI alone (0.1cc vs. 1.8cc, p<0.020). Similar results were found for EoR. (5-ALA&iMRI: 99.6% vs. iMRI 96.0% p<0.004). In iMRI cohort, two nPND (11%) and in 5-ALA &iMRI one nPND (5.6%) was found (p<1.0). Rate of complications was slightly higher in 5-ALA&iMRI group (5/18) as in iMRI group (3/18). The difference was not significant in Fisher´s test (p<0.691).

We tested whether there were associations between GTR and complications or nPNDs in both the iMRI and the 5-ALA&iMRI cohorts and in all 66 cases. No significant results were found (GTR & complication p< 0.617, GTR & nPND p< 0.121).

**Fig 2:**
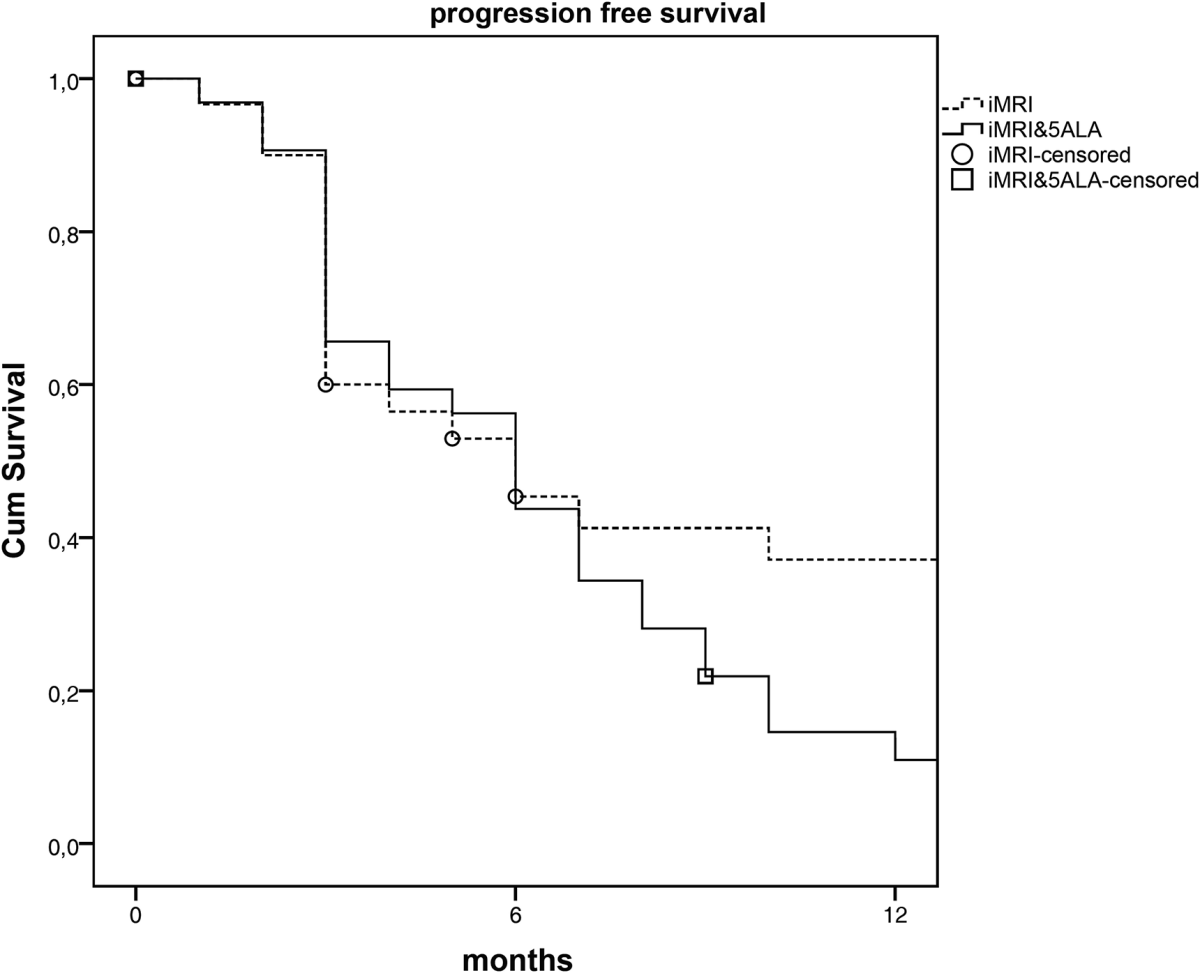
Kaplan Meier Plot of progression free survival by iMRI and 5-ALA&iMRI assisted surgery. iMRI intraoperative MRI; 5-ALA: 5 Aminolevulinic acid.

**Fig 3:**
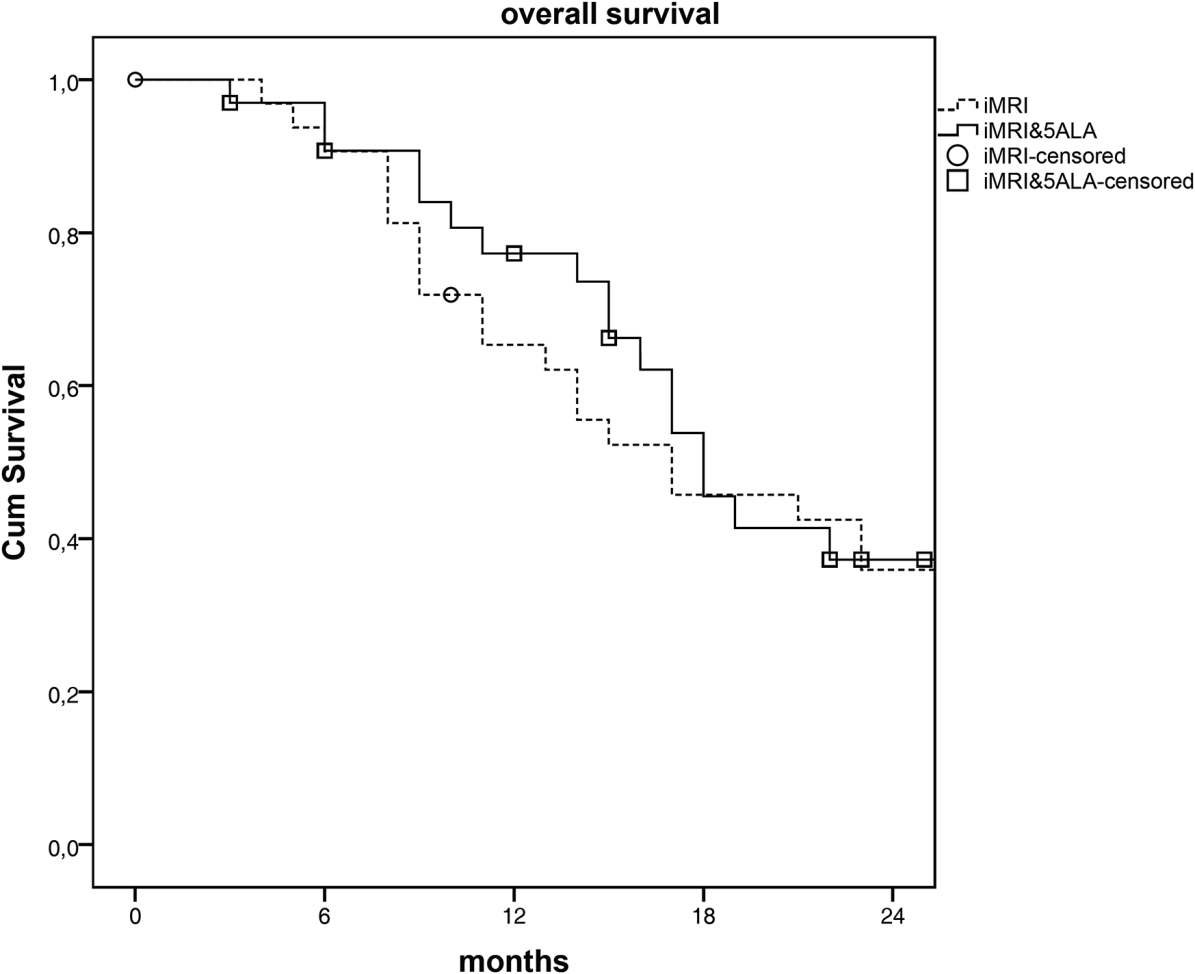
Kaplan Meier Plot of overall survival by iMRI and 5-ALA&iMRI assisted surgery. iMRI intraoperative MRI; 5-ALA: 5 Aminolevulinic acid.

Estimated median survival based on Kaplan Meyer estimates showed no difference for PFS (p<0.309) as well as OS (p<0.708). Median PFS was 6month in both groups. OS was similar too (18 vs. 17 months). Figs 2 and 3 show the survival curves based on PFS and OS for both groups. The curve of PFS shows no difference between both groups, while overall survival shows a tendency towards an increased survival in 5-ALA&iMRI group.

## Discussion

Diagnosis of a GBM is a terrible fate for the patient, despite years of research. From a surgical perspective, based on the previous literature, the best we can do is to remove as much tumor as possible without causing new neurological deficits.[6, 15] Apart from a precise definition of eloquent areas using brain mapping, intraoperative imaging has been shown to increase EoR. Basically, there are two approaches with class I evidence to support surgeons to achieve the goal of a GTR: 5-ALA fluorescence and intraoperative MRI. 5-ALA fluorescence improves tumor visualization and has been shown to improve EoR, and in consequence it was found to improve OS in the randomized controlled 5-ALA trial. [3] More recent data on low field iMRI demonstrated a significant increase on EoR and PFS in a prospective randomized series. [5] Especially the high-field iMRI technique allows for an intraoperative imaging similar to the postoperative resection control.[16] Gold standard for assessment of EoR and basis of response assessment in neuro-oncology (RANO) criteria is the Gd-DTPA enhancement. [17] On the other hand histopathological assessment as well as imaging studies have shown that the solid tumor exceeds Gd-DTPA enhancement. Obviously, there are additional sequences like diffusion, perfusion or molecular imaging that increase sensitivity in this regard.[18–21] However, large histological based studies are lacking so far. 5-ALA on the other hand has been shown to match better with metabolic active tumor in PET-CT than Gd-DTPA enhancement. [10, 22, 23] In low grade gliomas, a trend towards increased survival is shown for resection extending beyond the ‘visible’ tumor margins. [24] On the contrary, using 5-ALA close to eloquent areas might lead to an increase of nPND since surgeons might be guided outside of the planned safe margins. [25] In order not to cause neurological deficits surgeons might leave residual fluorescence due to anatomical uncertainties based on a lack of intraoperative update of imaging. Yet, this residual 5-ALA fluorescence, despite a GTR in postoperative MRI, was shown to be a negative predictor for PFS. [26] Based on intraoperative histopathological data on tumor depiction in 5-ALA and Gd-DTPA enhanced iMRI a synergistic effect of both imaging techniques can be presumed. [11] A combined imaging approach has been performed in two small series with promising results. [27, 28] Yet, no conclusion can be drawn whether there is an impact of the combined use on EoR and survival. Both, 5-ALA and iMRI have been used together with brain mapping in order to safely maximize EoR in eloquent areas [29, 30]. We also used neurophysiological monitoring and brain mapping techniques in all eloquent lesions routinely in the present series.

We provide a prospective series of patients who were operated using a combined approach of 5-ALA and iMRI for residual tumor control. We report a volumetric assessment of EoR and clinical outcome. The 5-ALA&iMRI cohort was compared to retrospective matched pairs of a historical control group who had surgery using iMRI alone before 5-ALA was introduced at our centerin order to prevent a selection bias. Thus, only patients with an intended GTR and similar MGMT promotor state, eloquent location, recurrent surgery, tumor size and age were compared. We found that a 5-ALA complete resection does not exclude a residual Gd-DTPA enhancement in postoperative MRI as described before.[27] Nevertheless, our data show that using 5-ALA as an adjunct to iMRI significantly increases the rate of EoR. Assessment of EoR in GBM is obviously based on postoperative Gd-DTPA enhancement. Thus, a resection beyond margins of contrast enhancement cannot be quantified in this approach. Unfortunately a volumetric assessment of the resection cavity also underestimates the amount of resected tissue. [31] Thus, we can only postulate based on our above mentioned histological data, that a resection beyond margins of contrast enhancement was achieved in gross-totally resected patients. Our data do not show any significant increase in complication or neurological deficits based on the combined imaging approach and a potential increase of EoR beyond margins of contrast enhancement. Yet, the significant increase of EoR in 5-ALA&iMRI group compared to iMRI alone did not result in an increase in PFS and OS. No trend can be derived from the survival data in our series. Even though the difference of an EOR of 97% (iMRI) vs. 100%(5-ALA&iMRI) was significant, it did not affect survival significantly in the small cohort (n = 66) reported here. With regard to the publication of Sanaii et al, who also found a stepwise improvement of survival even between EoR >95% up to 100%, we cannot exclude a type II error in our study. [32] However, even in the above mentioned study, the difference was seen according to Kaplan Meier estimates only in patients living longer than 20 months. Probably, only “long-term” survivors benefit from this increase of EoR.

Powerful studies are needed to assess impact of the combined use of 5-ALA&iMRI on survival. However, large series with n > 500 like those by the above mentioned authors are needed to assess such small effects. Unfortunately this will be rather unlikely to achieve. In our study sample sizes are decreased due to the matched pair assessment. Thus, a detailed subgroup assessment of eloquent or recurrent lesions is limited. From the insignificant results in recurrent disease we should not conclude that there is no benefit from a combined approach in these situations. From our surgical experience, especially in these cases 5-ALA was very helpful to differentiate scar tissue from recurrent tumor. Based on our case characteristics, we have to assume a typical cohort of GBM patients eligible for GTR generalizable to other centers. Yet, the retrospective matched pair control group potentially might lead to a selection bias. The decision for a GTR was based on the decision of the individual surgeon. Hence, a limitation of the study is the absence of an external blinded reviewer deciding for eligibility for GTR. Additionally no prospective randomization was performed due to limitations by the German pharmaceuticals act (AMG). The preoperative neurological status as an important confounding factor was not controlled in our series. However, no difference in nPND was found after surgery. Thus, a confounder in this regard might be insignificant. In our prospective study we compare only patients with lesions eligible for a GTR. The recent publication by Chaichana et al shows that there is not only a threshold for EoR to influence outcome, but also a residual volume below 5cc which significantly benefits survival.[33] Thus, the next step after having established a safe combined imaging approach in lesions eligible for GTR is to assess patients with intended STR.

In our present series, we used similar selection criteria as in the ‘5-ALA-trial’ by Stummer et al[3]. The median tumor volume in the 5-ALA trial was smaller and rate of eloquent locations lower as in our study. GTR was higher both in iMRI group (64% vs. 82%) and after combined imaging approach (64% vs 100%). A possible explanation might be the use of neurophysiological monitoring and brain mapping which was not the case in the ALA trial from 2006. The volumetric mean EoR of both iMRI and 5-ALA&iMRI group were above 97%. Especially, using the combined imaging approach the range of residual tumor (0–0.8cc) is distinctly smaller than previously published results for iMRI alone (0–4.7cc) and 5-ALA (0–26cc).[3, 34] In the prospective randomized trial by Senft et al on low-field iMRI a GTR rate of 96% was published for iMRI group. However, eloquent location was an exclusion criteria in this study. In another series including eloquent locations a GTR of 57% was found [5, 29] Hatiboglu et al performed a prospective volumetric assessment of EoR in high field iMRI similar to our study. [35] The authors provide a median EoR of 96% and a GTR of 71%, which is slightly lower than in our study. Roder et al published a retrospective series comparing iMRI assisted and 5-ALA based resections. [34] Results of iMRI assisted surgery is slightly lower, yet comparable to our series, while 5-ALA based resection was even lower than in the 5-ALA trial (46%). Resection rate of a contemporary series by Schucht et al. for 5-ALA and brain mapping report an EoR of 89% [30, 36]. Compared to actual literature, we show an additional increase of EoR combining 5-ALA and iMRI compared to iMRI and 5-ALA alone. This finding is supported by histological data of our group showing a distinctly different tumor depiction of 5-ALA fluorescence and Gd-DTPA enhancement at the border zone of GBMs. Thus, we can postulate a synergistic effect on tumor resection combining 5-ALA and iMRI. In order to safely maximizing EoR, neurophysiological monitoring and brain mapping are crucial. Thus, we recommend using these techniques when performing a combined approach. In this setup, we found no increase of nPND between iMRI alone and 5-ALA&iMRI. The rate of nPND in our series was lower than reported in the 5-ALA trial (6% vs.18%). Interestingly, when analyzing eloquent lesions only, the combined approach also achieved a significant higher EoR as using iMRI alone. Potentially the value of additional imaging might be limited close to eloquent areas. However, the contrary was found in our series. Additionally, rate of nPND stayed the same as in the whole series. As mentioned above, residual tumor close to eloquent areas is a negative prognosticator, which could be decrease in our series using a combined imaging approach with the adjunct of neurophysiological monitoring [33]. Actual publications using iMRI or 5-ALA alone and neurophysiological monitoring show nPND rates around 7%[30, 34] which are comparable to the rates in our series. Even though, we focused on maximizing EoR in the current study, our reported complication rate is lower than the reported cumulative complication rate of 29% on GBM surgeries.[37]

## Conclusion

We found a significant volumetric increase of extent of resection when combining 5-ALA and iMRI compared to use of iMRI alone. Maximizing EoR did not lead to an increase of complications or neurological deficits if used with neurophysiological monitoring in eloquent lesions. Mean EoR exceeded 97% in both groups. Due to this small difference in EoR in our data, no final conclusion can be drawn whether a further increase of EoR benefits patient’s progression free survival and overall survival.
